# Reduced Thiamine Availability and Hyperglycemia Impair Thiamine Transport in Renal Glomerular Cells through Modulation of Thiamine Transporter 2

**DOI:** 10.3390/biomedicines9040385

**Published:** 2021-04-05

**Authors:** Aurora Mazzeo, Federica Barutta, Linda Bellucci, Marina Trento, Gabriella Gruden, Massimo Porta, Elena Beltramo

**Affiliations:** 1 Department of Medical Sciences, University of Torino, 10126 Torino, Italy; aurora.mazzeo@unito.it (A.M.); federica.barutta@unito.it (F.B.); marina.trento@unito.it (M.T.); gabriella.gruden@unito.it (G.G.); massimo.porta@unito.it (M.P.); 2 Department of Molecular Biotechnology and Health Sciences, University of Torino, 10125 Torino, Italy; linda.bellucci@unito.it

**Keywords:** thiamine, renal glomerular cells, thiamine transporters, diabetic nephropathy, transketolase

## Abstract

Thiamine helps transketolase in removing toxic metabolites, counteracting high glucose-induced damage in microvascular cells, and progression of diabetic retinopathy/nephropathy in diabetic animals. Diabetic subjects show reduced thiamine levels. Hyperglycemia and reduced thiamine availability concur in impairing thiamine transport inside the blood-retinal barrier, with thiamine transporter-2 (THTR2) primarily involved. Here, we examined the behavior of thiamine transporter-1 (THTR1), THTR2, and their transcription factor Sp1 in response to high glucose and altered thiamine availability in renal cells involved in diabetic nephropathy. Human proximal tubule epithelial cells, podocytes, glomerular endothelial, and mesangial cells were exposed to high glucose and/or thiamine deficiency/oversupplementation. Localization and modulation of THTR1, THTR2, and Sp1; intracellular thiamine; transketolase activity; and permeability to thiamine were examined. Reduced thiamine availability and hyperglycemia impaired thiamine transport and THTR2/Sp1 expression. Intracellular thiamine, transketolase activity, and permeability were strongly dependent on thiamine concentrations and, partly, excess glucose. Glomerular endothelial cells were the most affected by the microenvironmental conditions. Our results confirmed the primary role of THTR2 in altered thiamine transport in cells involved in diabetic microvascular complications. Lack of thiamine concurs with hyperglycemia in impairing thiamine transport. Thiamine supplementation could represent a therapeutic option to prevent or slow the progression of these complications.

## 1. Introduction

The microvascular complications of diabetes are a primary cause of renal failure, blindness, and limb amputation in industrialized countries. Although their development has always been considered as strictly related to glycemic fluctuations and diabetes duration [1], a percentage of patients does not develop these complications despite poor glycemic control, while others, better controlled as regards glycated hemoglobin and blood pressure, are highly susceptible to them [2], suggesting the influence of other variables.

The role of thiamine in the prevention of diabetic complications is well acknowledged in the literature [3]. Thiamine is an essential cofactor in intracellular glucose metabolism, especially for transketolase (TK), an enzyme that shifts toxic metabolites from glycolysis to the pentose phosphate shunt [4,5]. Thiamine was shown to reduce reactive oxygen species (ROS) production in microvascular cells grown in diabetic-like conditions and in diabetic animals [6,7]. In vivo studies on animals with experimental diabetes showed that it reduces progression of diabetic nephropathy (DN) [8] and retinopathy (DR) [6]. Therefore, decreased thiamine availability may enhance metabolic damage, and diabetic patients often show reduced thiamine levels due to renal loss via proximal tubules, resulting in diminished TK activity [9].

Thiamine uptake inside the cells is regulated by two high-affinity transporters, THTR1 and THTR2, encoded respectively by the *SLC19A2* and *SLC19A3* genes [10], the levels of which vary in different tissues [11] and are regulated by the transcription factor Sp1 [12,13]. Compromised ability to reach intracellular thiamine concentrations able to match the increased glucose flux could account for susceptibility to the development of microvascular complications. In particular, insulin-independent cells, such as those involved in DN and DR, are unable to regulate glucose uptake, and therefore are more exposed to hyperglycemic damage [5].

Evidence in the literature shows downregulation of THTR1, THTR2, and Sp1 induced by high glucose in human kidney proximal tubular epithelium [14], while in human intestinal epithelial cells maintained in a low thiamine medium, thiamine uptake was upregulated, and THTR2 and Sp1 were overexpressed [15]. High glucose conditions and reduced thiamine availability synergically impair thiamine transport inside retinal cells and through the inner blood-retinal barrier. In particular, THTR2 expression is decreased in retinal pericytes, and increased in endothelial and Müller cells, suggesting a major role for THTR2 in thiamine transport in retinal cells, and its involvement in high-glucose-induced damage and impaired thiamine availability [3]. It was also demonstrated that two single-gene polymorphisms (SNP) located in the *SLC19A3* gene encoding for THTR2 are associated with resistance to the development of DR and DN, in subjects with long-term type 1 diabetes [16]. This suggests a role for genetic variations of THTR2 in the pathogenesis of diabetic microvascular complications, and a possible explanation for why some patients are less prone than others to diabetic complications.

Starting from these assumptions, our aims were to investigate the modulation of thiamine transporter expression in renal cells involved in DN and exposed to conditions mimicking the diabetic microenvironment, such as glucose fluctuations and thiamine deficiency, and if these variations could influence thiamine uptake and transketolase activity, ultimately worsening DN. Our results confirmed that THTR2 is the thiamine transporter more involved in DN, as we already demonstrated in DR [3], and that lack of thiamine concurs with hyperglycemia in impairing thiamine transport.

## 2. Materials and Methods

### 2.1. Cell Cultures

Primary human renal proximal tubule epithelial cells (HPTEC) were purchased from Lonza (Basel, Switzerland), cultured in Renal Epithelial Cell Growth Basal Medium (REGM, Lonza), and used at passage 6–9 for experiments. Conditionally immortalized human podocytes (HPC) [17] were kindly provided by M. A. Saleem, maintained in DMEM + 10% FCS at 33 °C for subculturing, and subsequently switched to 37 °C, where their growth arrests and they express podocyte markers. Human primary kidney glomerular endothelial cells (HGEC) were acquired from Cell Biologics (Chicago, IL, USA), grown in an EndoGRO-LS Complete Culture Media Kit (Merck, Darmstadt, Germany), and used at passage 6–9 for experiments. Immortalized human mesangial cells (HMC) [18], available in our department, were cultured in DMEM + 10% FCS. All cell types were switched to DMEM + 10% FCS for experiments. As regards the experimental setting, cells were cultured for 8 days in physiological D-glucose concentrations (NG, 5.6 mmol/L), stable high glucose (HG, 28 mmol/L), or intermittent high/physiological glucose (intHG, 48 h HG/48 h NG, twice), to mimic the diabetic microenvironment. Moreover, to investigate the substrate influence on the expression of thiamine transporters, thiamine was added at a pharmacological dose of 50 µmol/L (HT), while custom-made thiamine-deficient media (TD) (Thermo Fisher Scientific, Waltham, MA, USA) was used to test the effects of low thiamine environment.

To study the interactions among different cell types, we also used two different types of cocultures, by growing HGEC on the inner membrane surface of transwell inserts, and either HPC or HMC on the bottom of the well. Cocultures were maintained for 8 days in the above-described experimental conditions. At the end of the incubation period, inserts were shifted to new wells; trypsin-EDTA was added inside the insert to detach HGEC, while HPC or HMC were collected by trypsinization from the bottom of the original wells.

### 2.2. Localization of Thiamine Transporters THTR1/2 and Sp1

Cells cultured as described above were transferred to chamber slides for the last 24 h. They were then fixed in ice-cold methanol for 5 min at −20 °C, dried at RT for 15 min, and rehydrated in PBS for 15 min. Nonspecific binding sites were blocked in PBS plus 0.2% BSA (blocking solution) for 1 h at RT. Cells were incubated overnight at 4 °C with 1 µg/mL rabbit polyclonal anti-SLC19A2 (Merck Cat# HPA006119, RRID:AB_1079996), 2 µg/mL rabbit polyclonal anti-SLC19A3 (Merck Cat# HPA038898, RRID:AB_10673481), or 1:250 rabbit polyclonal anti-SP1 (Thermo Fisher Scientific Cat# PA5-27243, RRID:AB_2544719), as appropriate. Following three washings with blocking solution, 1:1000 FITC-conjugated goat anti-rabbit IgG (Abcam Cat# ab6717, RRID:AB_955238) was added for 1 h at RT. DAPI was used to blue-stain the cell nuclei. Images were taken under a Leica DM 2000 microscope (Leica, Wetzlar, Germany) equipped with a Leica DFC 320 camera and Leica QWin Plus 2003 digital processing and analysis software.

### 2.3. Modulation of the Expression of Thiamine Transporters THTR1/2, Sp1, and TK

#### 2.3.1. Quantitative Real Time PCR (qRT-PCR)

THTR1/2, Sp1, and TK mRNA expression were evaluated by qRT-PCR. Total RNA was extracted with the HighPure RNA Isolation kit (Merck), and 200 ng RNA were reverse-transcribed using the High Capacity cDNA Reverse Transcription kit (Thermo Fisher Scientific). The qRT-PCR was performed by 48-well StepOne Real Time System (Applied Biosystems, Foster City, CA, USA), using Power SYBR™ Green PCR Master Mix (Thermo Fisher Scientific). Relative gene expression was determined using the 2^−ΔΔCT^ method and normalized against β-actin. Primers used were: THTR1 forward: 5′-AGCCAGACCGTCTCCTTGTA-3′, reverse: 5′-TAGAGAGGGCCCACCACAC-3′; THTR2 forward: 5′-CTGGCTCTGGTGGTCTTCTC-3′, reverse: 5′-AGGCATAGCGTTCCACATTC-3′; Sp1 forward: 5′-TGCAGCAGAATTGAGTCACC-3′, reverse: 5′-CACAACATACTGCCCACCAG-3′ [3]; TK forward: 5′-CCCAGCTACAAAGTTGGGGACAAG-3′; reverse, 5′-GGTCATCCTTGCTCTTCAGGACC-3′.

#### 2.3.2. Western Blot Analysis

THTR1/2 and Sp1 protein expression were evaluated by Western blot analysis. To extract total proteins, cells were lysed using M-PER Mammalian Protein extraction reagent (Thermo Fisher Scientific) added with 10 µL/mL protease inhibitor cocktail kit (Thermo Fisher Scientific). Extracts were kept ice-cold and cleared by centrifugation at 20,000× *g* for 15 min at 4 °C. The supernatant was aliquoted and stored at −80 °C. Protein content was measured through the Bradford reaction. A total of 30 µg of proteins was loaded on precast gels (4–20% Mini-PROTEAN^®^ TGX™ Precast Gel, Bio-Rad, Hercules, CA, USA), separated by electrophoresis, and transferred to nitrocellulose membranes. Immunoblotting was performed by incubating the membranes with rabbit polyclonal antibody anti-SLC19A2 (Abcam Cat# ab123246, RRID:AB_10972351) 1:1000, anti-SLC19A3 (Abcam Cat# ab103950, RRID:AB_10711742) 1:500, and anti-Sp1 (Thermo Fisher Scientific Cat# PA5-27243, RRID:AB_2544719) 1:2000. Immunoreactive bands were visualized using the enhanced chemiluminescence (ECL) Western blotting protocol (Merck). The relative signal strength was quantified by densitometric analysis (1D Image Analysis System, Kodak), and values were normalized against β-actin (Sigma-Aldrich, Cat#A5316, RRID:AB_476743) or vinculin (Sigma-Aldrich, Cat# V9131, RRID:AB_477629), as appropriate.

### 2.4. Intracellular Thiamine Uptake

Intracellular thiamine uptake was measured in cell lysates through the DRG^®^ Vitamin B1 (Thiamine) (BIO-5136) kit (DRG International, Springfield, NJ, USA). A total of 150 µL of 1:5 diluted lysate and 150 µL medium from the kit were added to 96-well plates coated with *Lactobacillus fermentum*. The presence of thiamine in samples gave a thiamine-dependent growth response until the vitamin is consumed. After incubation at 37 °C for 48 h, the growth of *L. fermentum* was measured turbidimetrically at 630 nm. Thiamine concentration was directly proportional to the turbidity. Values were normalized by protein content.

### 2.5. Transketolase Activity

Transketolase activity was evaluated using a modification of the enzymatic kinetic method of Chamberlain et al. [19], as previously described [3]. The enzyme reaction was started by the addition of 15 µL cell lysate to 85 µL reaction mixture (15 mmol/L ribose-5-phosphate, 250 µmol/L NADH, 0.1 mol/L Tris-HCl pH 7.8, 200 U/mL glycerol-3-phosphate dehydrogenase/triose phosphate isomerase), and the 340 nm absorbance was measured at 10 min intervals for 90 min. TK activity was deduced from the difference in the absorbance at t = 0 and t = 30 min for HMC and HPTEC, and t = 0 and t = 60 min for HPC and HGEC. Values were normalized by protein content.

### 2.6. Permeability to Thiamine of HPC/HGEC Bilayers

Permeability to thiamine was measured by growing HPC on the inner membrane surface, and HGEC on the outer surface, of transwell inserts. Once confluence was reached, the medium was changed to thiamine-deficient medium, and 50 µmol/L thiamine were added to the upper chamber and thiamine content measured in the lower chamber after 1 hr incubation, as described above.

### 2.7. Statistics

Statistical comparison was performed by one-way ANOVA with Bonferroni post hoc correction. Results were expressed as mean ± SD of 5 independent experiments, as previously established through a power analysis, and normalized against cells cultured in physiological conditions (NG).

## 3. Results

### 3.1. Localization and Expression of Thiamine Transporters and Sp1 in Renal Cells in Physiological Conditions

**Figure 1:**
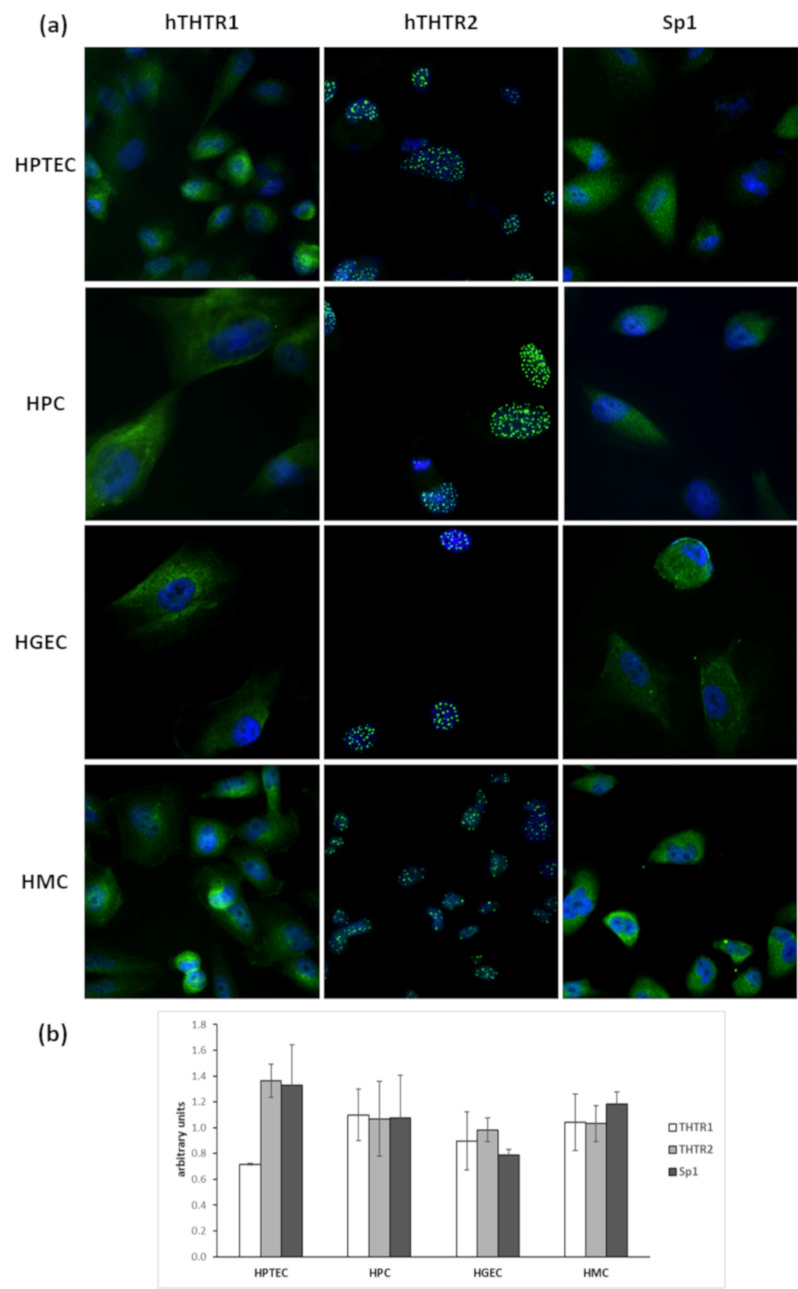
Localization and expression of thiamine transporters and Sp1 inside renal glomerular cells in physiological conditions. (**a**) Immunofluorescence with Ab anti-THTR1, anti-THTR2, and anti-Sp1 in HPTEC, HPC, HGEC, and HMC (green). Nuclei are counterstained with DAPI (blue). Magnification 400×. (**b**) Relative expression of THTR-1, THTR2, and Sp1 in the 4 cell types. Mean of 5 experiments ± SD.

All examined renal glomerular cell types expressed the two thiamine transporters and their transcription factor Sp1 in physiological conditions (Figure 1). THTR1 and Sp1 showed a cytoplasmic distribution in all cell types at the immunofluorescence staining, while THTR2 located inside the cell nucleus, more precisely in nuclear cell bodies (Figure 1a). In HPC, HGEC, and HMC, the relative concentrations of the three factors were comparable, while in HPTEC, THTR1 was less expressed than THTR2 and Sp1 (Figure 1b).

### 3.2. Expression of Thiamine Transporters and Sp1 in Cocultured Renal Cells under Different Glucose Conditions

**Figure 2:**
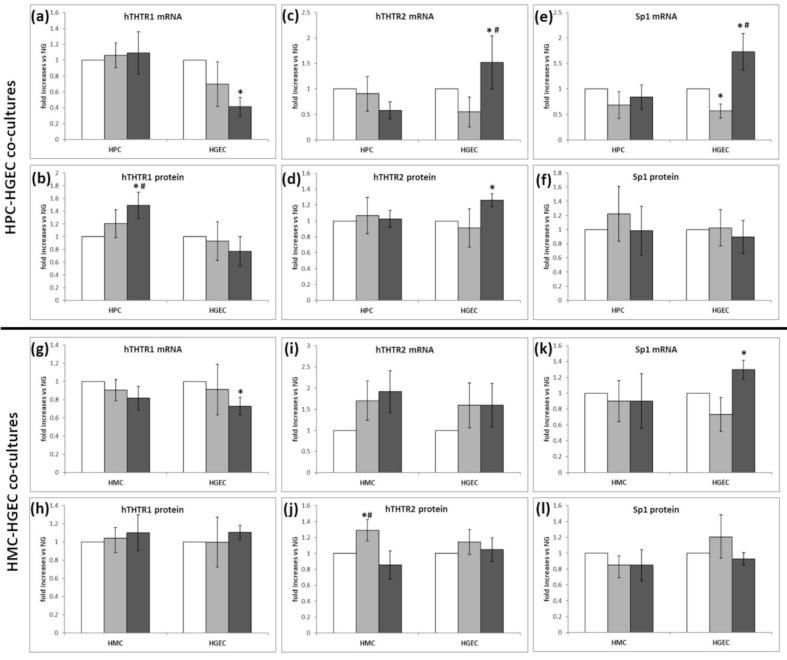
THTR1, THTR2 and Sp1 expression in cocultured renal glomerular cells under different experimental conditions. (**a**–**f**) HPC–HGEC cocultures, (**g**–**l**) HMC–HGEC cocultures. (**a**,**g**) THTR1 mRNA and (**b**,**h**) protein expression; (**c**,**i**) THTR2 mRNA and (**d**,**j**) protein expression; (**e**,**k**) Sp1 mRNA and (**f**,**l**) protein expression. White bars: physiological glucose concentrations (NG); light grey bars: high glucose concentrations (HG); dark grey bars: intermittent high glucose conditions (intHG). NG = physiological glucose concentrations; HG = high glucose concentrations; intHG = intermittent high glucose concentrations. Mean of 5 experiments ± SD. * *p* < 0.05 vs. NG; # *p* < 0.05 vs. HG.

Two different models were created to mimic the glomerular microenvironment: HPC/HGEC and HMC/HGEC cocultures on transwell inserts. Cells were exposed to physiological, high, or intermittent high/low glucose concentrations, to mimic the diabetic microenvironment, and mRNA and protein expression of thiamine transporters and Sp1 were determined (Figure 2). THTR1 expression was substantially unchanged in all cell types and both coculture models, apart from increased protein expression in HPC in intHG (Figure 2b), and decreased mRNA expression in HGEC in intHG, present in both coculture models (Figure 2a,g). THTR2 showed no differences in expression in HPC (Figure 2c,d), while it increased both mRNA and protein expression in HGEC cocultured with HPC after exposure to intHG. THTR2 protein expression in HMC was increased in HG conditions (Figure 2j). Sp1 mRNA expression was increased in HGEC in both coculture systems (Figure 2e,k).

### 3.3. Expression of Thiamine Transporters and Sp1 in Renal Cells under Different Experimental Conditions

All cell types were cultured in the above-described glucose conditions (NG, HG, intHG), while the influence of substrate (thiamine) on the expression of its transporters was investigated by adding thiamine at pharmacological doses (HT), or using thiamine-deficient media (TD), together with each glucose condition. The mRNA and protein expression of each transporter and Sp1 were evaluated in all experimental conditions.

**Figure 3:**
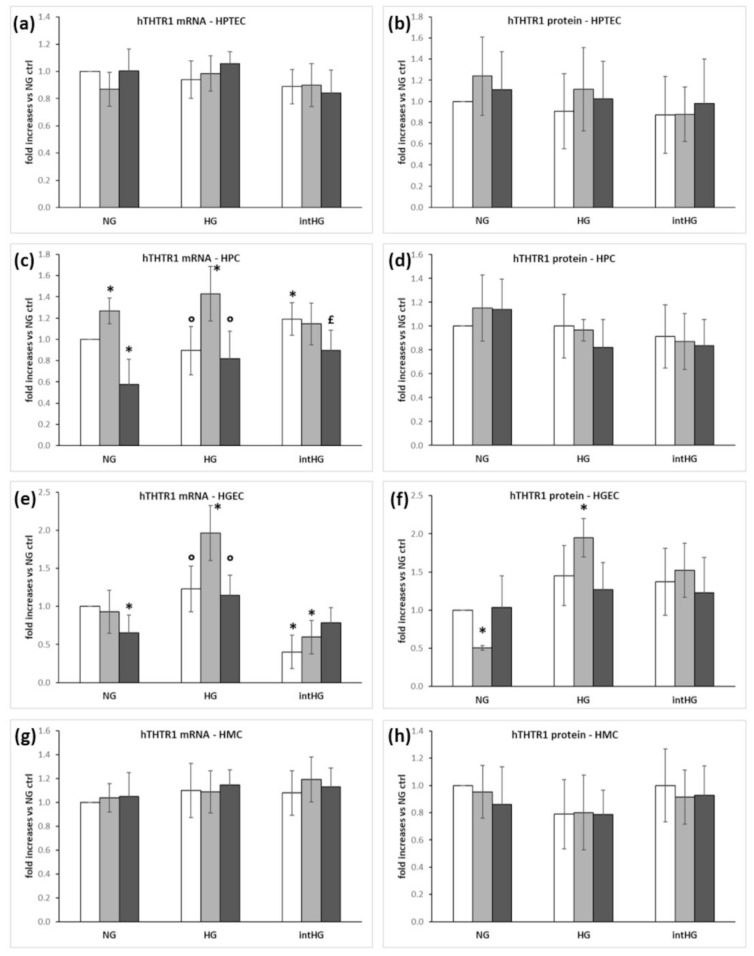
THTR1 expression in renal glomerular cells under different experimental conditions. (**a**) THTR1 mRNA and (**b**) protein expression in HPTEC; (**c**) THTR1 mRNA and (**d**) protein expression in HPC; (**e**) THTR1 mRNA and (**f**) protein expression in HGEC; (**g**) THTR1 mRNA and (**h**) protein expression in HMC. White bars: physiological thiamine concentrations (ctrl); light grey bars: thiamine-deficient medium (TD); dark grey bars: high thiamine concentrations (HT). NG = physiological glucose concentrations; HG = high glucose concentrations; intHG = intermittent high glucose concentrations. Mean of 5 experiments ± SD. * *p* < 0.05 vs. NG ctrl; ° *p* < 0.05 vs. HG TD; £ *p* < 0.05 vs. intHG ctrl and intHG TD.

THTR1 mRNA and protein expressions were unchanged in HPTEC (Figure 3a,b) and HMC (Figure 3g,h) in all experimental conditions. In HPC, THTR1 mRNA (Figure 3c), but not protein (Figure 3d), increased in intHG and TD conditions, while in HGEC, the TD condition increased both THTR1 mRNA and protein expression (Figure 3e,f).

**Figure 4:**
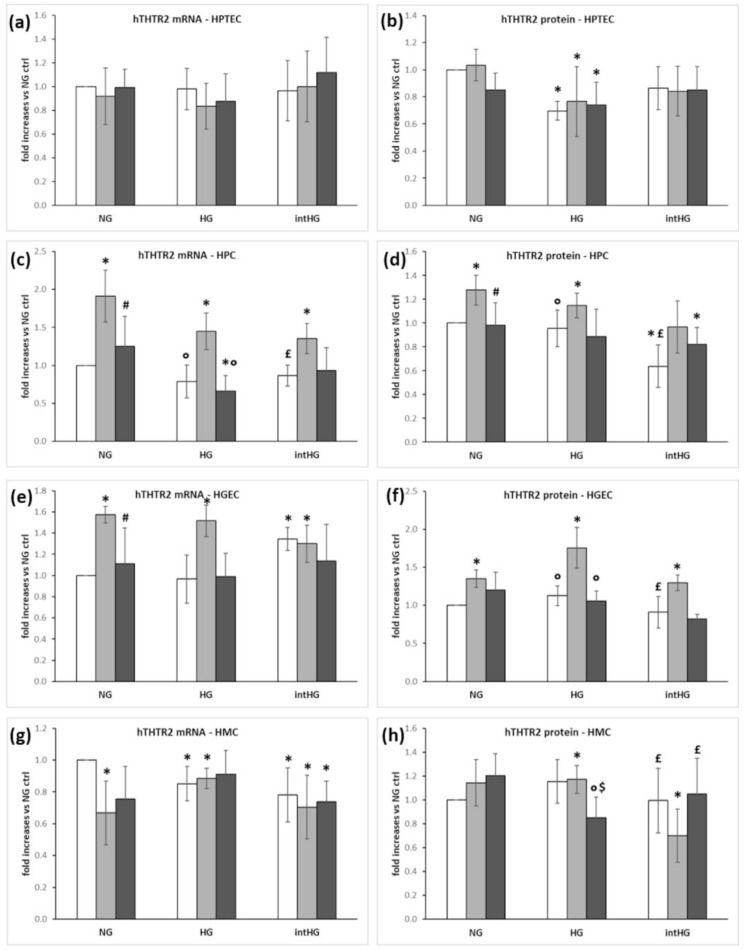
THTR2 expression in renal glomerular cells under different experimental conditions. (**a**) THTR2 mRNA and (**b**) protein expression in HPTEC; (**c**) THTR2 mRNA and (**d**) protein expression in HPC; (**e**) THTR2 mRNA and (**f**) protein expression in HGEC; (**g**) THTR2 mRNA and (**h**) protein expression in HMC. White bars: physiological thiamine concentrations (ctrl); light grey bars: thiamine-deficient medium (TD); dark grey bars: high thiamine concentrations (HT). NG = physiological glucose concentrations; HG = high glucose concentrations; intHG = intermittent high glucose concentrations. Mean of 5 experiments ± SD. * *p* < 0.05 vs. NG ctrl; # *p* < 0.05 vs. NG TD; ° *p* < 0.05 vs. HG TD; £ *p* < 0.05 vs. intHG TD; $ *p* < 0.05 vs. HG ctrl.

THTR2 protein expression was 20% decreased in HPTEC in HG, independently of thiamine concentration (Figure 4b), while mRNA expression was unchanged (Figure 4a). HG and intHG also seemed to be associated with THTR2 decrease in HMC (Figure 4g–h). On the contrary, in HPC and HGEC, it was thiamine deficiency, rather than glucose concentrations, that determined a 50–60% increase in both THTR2 mRNA/protein expressions (Figure 4c–f).

**Figure 5:**
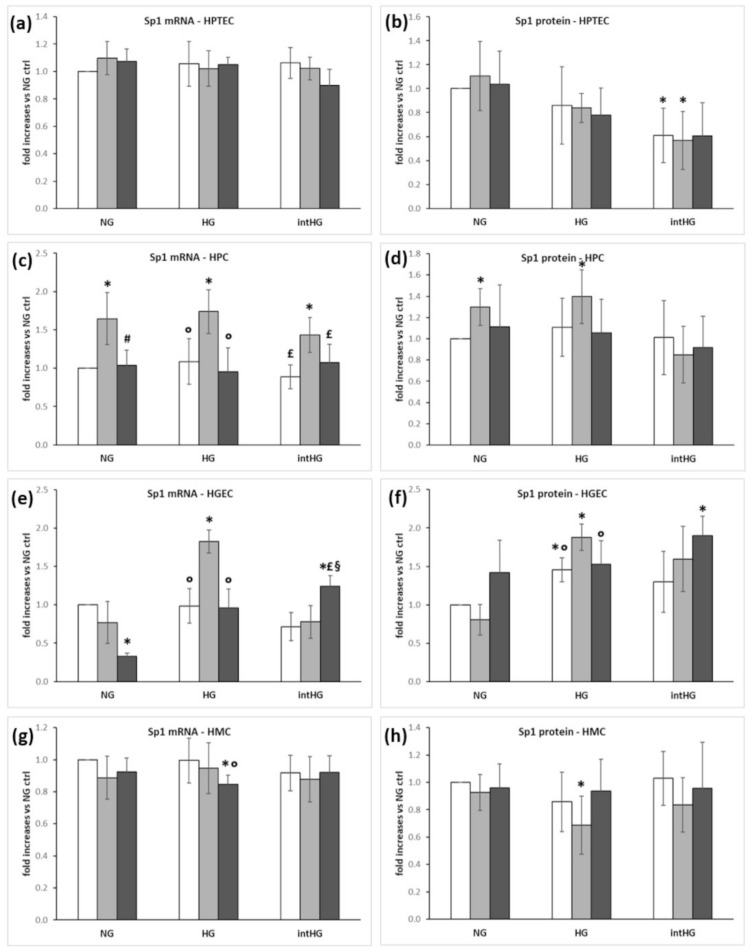
Sp1 expression in renal glomerular cells under different experimental conditions. (**a**) Sp1 mRNA and (**b**) protein expression in HPTEC; (**c**) Sp1 mRNA and (**d**) protein expression in HPC; (**e**) Sp1 mRNA and (**f**) protein expression in HGEC; (**g**) Sp1mRNA and (**h**) protein expression in HMC. White bars: physiological thiamine concentrations (ctrl); light grey bars: thiamine-deficient medium (TD); dark grey bars: high thiamine concentrations (HT). NG = physiological glucose concentrations; HG = high glucose concentrations; intHG = intermittent high glucose concentrations. Mean of 5 experiments ± SD. * *p* < 0.05 vs. NG ctrl; # *p* < 0.05 vs. NG TD; ° *p* < 0.05 vs. HG TD; £ *p* < 0.05 vs. intHG TD; § *p* < 0.05 vs. intHG ctrl.

Sp1 expression was unchanged in HPTEC and HMC, with an influence of intHG concentration on HPTEC Sp1 protein (Figure 5a,b,g,h), while in HPC and HGEC, TD deficiency increased Sp1 expression (Figure 5c–f).

### 3.4. Intracellular Thiamine Uptake, Transketolase Activity, and Expression in Renal Cells

**Figure 6:**
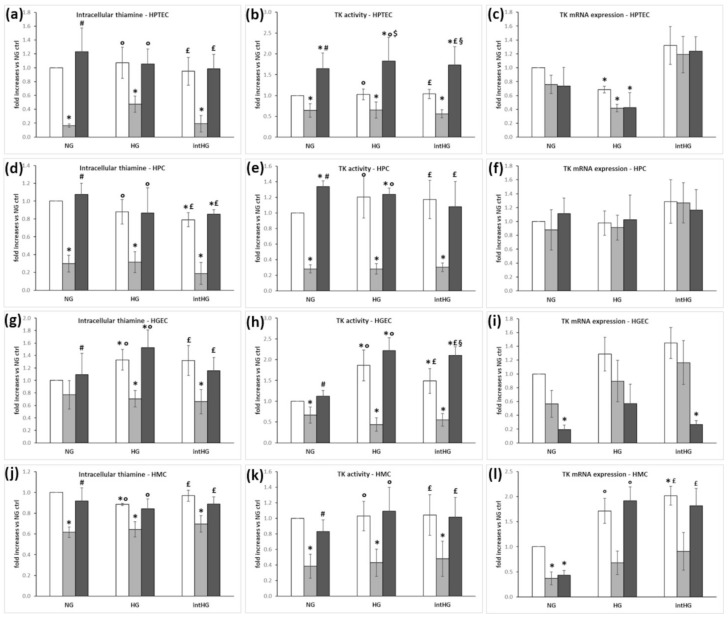
Intracellular thiamine concentration, TK activity, and TK mRNA expression in renal cells under different experimental conditions. (**a**) Intracellular thiamine, (**b**) TK activity, and (**c**) TK mRNA expression in HPTEC; (**d**) intracellular thiamine, (**e**) TK activity, and (**f**) TK mRNA expression in HPC; (**g**) intracellular thiamine, (**h**) TK activity, and (**i**) TK mRNA expression in HGEC; (**j**) intracellular thiamine, (**k**) TK activity, and (**l**) TK mRNA expression in HMC. White bars: physiological thiamine concentrations (ctrl); light grey bars: thiamine-deficient medium (TD); dark grey bars: high thiamine concentrations (HT). NG = physiological glucose concentrations; HG = high glucose concentrations; intHG = intermittent high glucose concentrations. Mean of 5 experiments ± SD. * *p* < 0.05 vs. NG ctrl; # *p* < 0.05 vs. NG TD; ° *p* < 0.05 vs. HG TD; £ *p* < 0.05 vs. intHG TD.

Intracellular thiamine uptake, TK activity, and expression after exposure to all the different experimental conditions were assessed as indirect measures of thiamine transporter function. TD was associated with changes in intracellular thiamine concentrations and TK activity: we found remarkable decreases in both parameters, independent of glucose concentration, in all cell types (Figure 6a,b,d,e,g,h,j,k). In addition, HGEC also were susceptible to HG and intHG concentrations, with upregulation of intracellular thiamine and TK activity (Figure 6g,h). In HPC, TK activity, but not thiamine intracellular concentration, increased in HG and intHG conditions (Figure 6d,e). HT concentrations increased TK activity in HPTEC (Figure 6b). As regards TK expression, it was substantially unaffected by TD in all cell types (Figure 6c,f,i), except for mesangial cells, in which it decreased similarly to intracellular thiamine concentration and TK activity (Figure 6l). TK expression also diminished in HPTEC cultured in HG concentrations (Figure 6c), and in HGEC in high thiamine medium (Figure 6i).

### 3.5. Permeability to Thiamine of HPC/HGEC Cocultures

**Figure 7:**
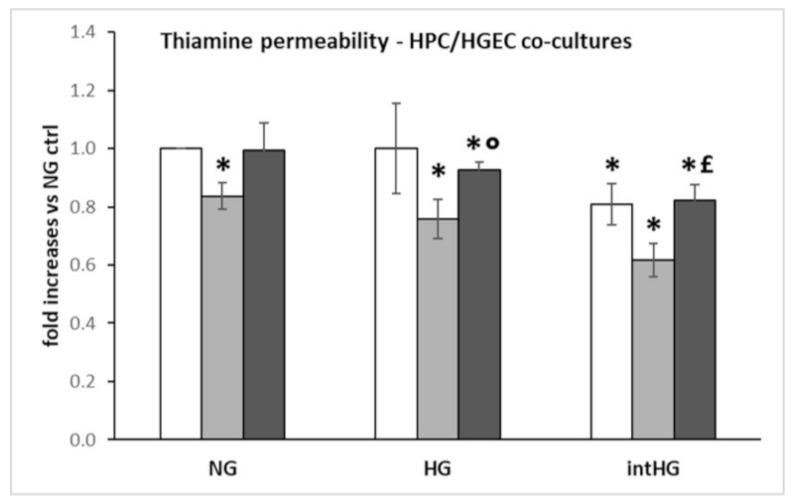
Thiamine permeability in HPC/HGEC cocultures. White bars: physiological thiamine concentrations (ctrl); light grey bars: thiamine-deficient medium (TD); dark grey bars: high thiamine concentrations (HT). NG = physiological glucose concentrations; HG = high glucose concentrations; intHG = intermittent high glucose concentrations. Mean of 5 experiments ± SD. * *p* < 0.05 vs. NG ctrl; ° *p* < 0.05 vs. HG TD; £ *p* < 0.05 vs. intHG TD.

Thiamine filtration through HPC/HGEC confluent bilayers previously exposed to all experimental conditions was decreased in HPC/HGEC cocultures previously cultured in TD conditions, regardless of glucose concentration. The same effect was found in cultures pretreated with intHG (Figure 7).

## 4. Discussion

Our findings suggest that reduced thiamine availability concurs with hyperglycemia to impair thiamine transport into cells involved in DN, with THTR2 primarily involved. In this work, three cell types belonging to the human renal glomerulus (podocytes, glomerular endothelial cells, and mesangial cells), together with human proximal tubule epithelial cells, were utilized in order to give a comprehensive view of all the actors interested in this complication.

The distribution and relative expression of thiamine transporters is known to differ throughout the body [11], with THTR1 and Sp1 rather ubiquitous, and THTR2 absent in certain locations, such as pancreatic beta cells, marrow stem cells, and cochlear hairy cells [14,20,21]. We demonstrated the presence of both transporters and Sp1 in retinal cells of the inner blood-retinal barrier, with Sp1 relative concentration being the highest, and THTR1 more expressed than THTR2 [3]. All cell types we examined expressed both thiamine transporters and Sp1; THTR1 was less expressed than the other two factors in HPTEC only. THTR1 and Sp1 were widely distributed into the cytoplasm, while, rather surprisingly, THTR2 was localized inside the nucleus. Although this finding is compatible with the protein compartmentation described in the database GeneCards (https://www.genecards.org), and consistent with our previous findings on human retinal pericytes and Müller cells [3], its meaning remains difficult to understand, and would probably be worthy of further investigation. The results obtained with renal glomerular/proximal tubule cells were superimposable to those of retinal cells in terms of expression and intracellular distribution of thiamine transporters, and were stable in all glucose/thiamine conditions examined. Thiamine transporter localization had been already addressed in proximal tubule epithelial cells [14]; however, this is the first time, to our knowledge, that it has been studied in renal glomerular cells.

Evidence in the literature demonstrates different modulations of the expression of thiamine transporters in cells from different districts, in response to variations in glucose or thiamine concentrations. Decreased expression of THTR1 and THTR2 in human proximal tubule epithelial cells in response to high glucose concentrations was described [14], as well as diminished THTR2 in HUVEC [22] and retinal pericytes [3], while endothelial and Müller cells showed the opposite behavior [3]. Thiamine deficiency, on the other hand, upregulated THTR2 expression in intestinal epithelial barrier Caco-2 cells [15] and retinal epithelial ARPE-19 cells [23], but not in cells from the inner blood-retinal barrier [3]. This suggests cell-specific susceptibility, similar to what reported in other diseases: THTR2 was shown downregulated in Sprague–Dawley rats with chronic kidney disease [24], upregulated in breast cancer [25], and unchanged in erythrocytes of diabetic subjects, in comparison with healthy controls [22].

The effects of different glucose conditions on the expression of thiamine transporters and Sp1 were firstly examined in coculture models of renal glomerular cells. Glomerular endothelial cells come in contact with both podocytes and mesangial cells at different sites; therefore, we created two different coculture models to evaluate if these interactions could play a role in the modulation of thiamine transporters in a diabetic-like microenvironment. Since previous evidence showed that human retinal microvascular cells are more sensitive to fluctuating than stable high glucose concentrations [26,27], consistently with the daily glycemic fluctuations in diabetic subjects, we chose to test both stable and intermittent high glucose concentrations on renal cells. Our results showed that THTR1 was slightly influenced by glucose variations in either model, while THTR2 and Sp1 expression increased in HGEC and, partly, in HMC, under high/intermittent glucose conditions, probably as a consequence of the increased need of thiamine inside the cells to deal with the excess glucose concentrations and accelerated glycolytic flux. Even though THTR1 and THTR2 may be found in almost all districts of the body, defects in their expression may assume different meanings. A rare form of diabetes associated with thiamine-responsive megaloblastic anemia (TRMA) is caused by a defect in *SLC19A2* gene, encoding for THTR1 [28,29], while THTR2 truncated proteins as a result of mutations in *SLC19A3* gene provoke biotin-responsive basal ganglial disease [30] and thiamine-responsive encephalopathy [31]. Our present results, together with previous data on cells of the inner blood-retinal barrier [3], and the evidence that two SNPs located in the *SLC19A3* gene are strongly associated with absent or minimal DR and DN [16], account for a primary involvement of THTR2 in diabetes microvascular complications.

Thiamine deficiency is associated with diabetes: diabetic patients frequently show low thiamine levels and reduced TK activity due to renal loss through the proximal tubule [9], and this may facilitate metabolic damage. Thiamine supplementation to diabetic patients has been shown to help in studies addressing DN and neuropathy [32], but larger trials are needed to confirm these observations. To better understand the role of thiamine and its deficiency in our diabetic-like milieu, all cell types were exposed to three different thiamine concentrations: physiological (ctrl), low (TD), and pharmacological (HT), in combination with the three glucose conditions. THTR1 expression substantially confirmed the very low variations we found in cocultured models in different glucose concentrations, while thiamine deficiency combined with HG increased THTR1 expression in HGEC and HPC. This is consistent with evidence reporting increased THTR1 expression in ARPE-19 cells, inversely correlated with extracellular thiamine concentrations [23]. As previously shown in retinal models [3], THTR2 appears to be the most susceptible of the three factors to the metabolic changes in the microenvironment, but with considerable differences among cell types. While altered glucose concentrations account mainly for decreased THTR2 expression in HPTEC and HMC, thiamine deficiency resulted in increased THTR2 in HPC and HGEC. This marks a difference with our results in retinal cells, where THTR2 expression increased in endothelial and Müller cells, and decreased in pericytes, as a consequence of glucose overexposure, but was not influenced by thiamine concentrations in the medium [3]. The different behavior of HPTEC and HMC on one side, and HPC and HGEC on the other, also was maintained as regards Sp1 expression, which was unchanged in the former, and upregulated by thiamine deficiency in the latter. These results demonstrate a greater involvement of the thiamine-deficiency status in the modifications of thiamine transporter expression in renal glomerular cells, in contrast with retinal cells, which are less disturbed by thiamine levels, but more sensitive to glucose fluctuations.

Intracellular thiamine concentrations and transketolase activity were mainly dependent on thiamine concentration in the medium, as expected. Thiamine deficiency reflected directly in low intracellular concentrations, and this, in turn, led to a reduction in TK activity, evidencing the strong dependency of TK on its coenzyme. On the other hand, thiamine deficiency does not influence TK expression in proximal tubule epithelial cells, podocytes, and glomerular endothelial cells, thus confirming that in these cells, the decreased TK activity depends mainly on intracellular thiamine concentration. Indeed, in HGEC high thiamine concentrations account for a decrease in TK expression and a concomitant increase in its activity. In mesangial cells, on the contrary, the trend shown by TK expression was very similar to that of TK activity; we can therefore suppose that in this particular cell type, thiamine deficiency may affect TK expression, rather than its activity. In HPTEC, increased thiamine availability in the substrate led to amplified TK activity. Moreover, high and intermittent glucose conditions enhanced thiamine uptake and, consequently, TK activity in HGEC. As this does not appear to be related to increased thiamine transporter expression in the same conditions, it could be explained by greater thiamine request in response to augmented glucose intake.

Finally, we measured the permeability to thiamine of podocytes/HGEC bilayers, after previous exposure of the cells to different experimental conditions. We found decreased permeability in cells cultured in thiamine-deficient media, and in all intermittent high-glucose conditions. These results are superimposable on our previous findings in retinal cells [3], and can be explained with retainment inside the cell of all available vitamin, to counteract thiamine starvation and hyperglycemic damage [5].

The similar results obtained with retinal cells of the inner blood-retinal barrier and the renal cells examined in this work are of interest, because all of them are insulin-independent and unable to regulate glucose uptake. Thus, they are primarily interested by the hyperglycemic damage, and prone to complications, such as DR and DN. Susceptibility to develop these complications may be related to a reduced ability to reach sufficient intracellular thiamine concentrations to eliminate toxic metabolites derived from increased glucose uptake [4]. In fact, thiamine is an essential coenzyme for TK, which shifts excess metabolites from glycolysis toward alternative pathways, and for enzymes involved in the subsequent Krebs cycle.

A limit of this work is the use of cell cultures only. Extrapolating cells from the complexity of the human organism to isolate a single phenomenon has its obvious limitations. However, it allows a better understanding of the involvement of the single cell type. We chose to use site-specific human cells only, to rule out the possible issues and misinterpretations due to the use of cells from other species or locations. To our knowledge, this is the first report addressing the role of thiamine transporters and thiamine deficiency in all cell types involved in DN. A future development of this study could address the potential role of genetically determined changes of thiamine transporters in diabetic microangiopathy, to better understand its pathogenesis on one hand and, by developing simple test procedures, to identify those diabetic subjects who are less or more at risk of developing complications at an early stage.

## 5. Conclusions

In conclusion, reduced thiamine availability, alone or together with high glucose conditions, impairs glomerular thiamine transport. THTR2, together with its transcription factor Sp1, is primary involved in high-glucose-induced damage and altered thiamine availability. This confirms our previous findings on retinal cells of the inner blood-retinal barrier [3], and the relevance of genetic mutations of the *SLC19A3* gene encoding for THTR2 in the resistance to the development of DR and DN in subjects with long-term type 1 diabetes [16]. As regards the cell types under study, endothelial cells seemed to be most affected by the altered microenvironmental conditions at the glomerular level, in terms of impaired intracellular thiamine transport.

In the light of these findings and literature reports about the beneficial effects of thiamine in both in vitro and in vivo studies, thiamine supplementation would be a reasonable option to prevent or slow the progression of microvascular complications in diabetic patients. However, appropriate clinical studies are still lacking, and will surely be needed to further explore the potential of thiamine.

## Data Availability

Data will be made available by the corresponding author upon reasonable request.

## Abbreviations

DN	diabetic nephropathy
DR	diabetic retinopathy
HG	high D-glucose concentrations
HGEC	human glomerular endothelial cells
HMC	human mesangial cells
HPC	human podocytes
HPTEC	human renal proximal tubule epithelial cells
HT	high thiamine concentrations
intHG	intermittent high D-glucose concentrations
NG	physiological D-glucose concentration
ROS	reactive oxygen species
SNP	single gene polymorphism(s)
Sp1	transcription factor specificity protein-1
TD	thiamine deficiency/deficient
THTR1	thiamine transporter-1
THTR2	thiamine transporter-2
TK	transketolase
TRMA	thiamine-responsive megaloblastic anemia
